# The relationship between positive psychological qualities and prenatal negative emotion in pregnant women: A path analysis

**DOI:** 10.3389/fpsyg.2022.1067757

**Published:** 2023-01-05

**Authors:** Xiabidan Tuxunjiang, Gulijianati Wumaier, Wei Zhang, Bahedana Sailike, Xiaoting Wang, Ting Jiang

**Affiliations:** ^1^Department of Public Health, Xinjiang Medical University, Ürümqi, China; ^2^Xinjiang Production and Construction Corps 13th Division Red Star Hospital, Hami, China

**Keywords:** pregnant women, prenatal depression, prenatal anxiety, positive psychological quality, path analysis

## Abstract

**Objective:**

The objective of this study was to investigate the relationship between positive psychological qualities and negative emotions of pregnant women.

**Methods:**

We surveyed 774 pregnant women in a tertiary hospital in Urumqi using the following measures: a self-report general demographic data questionnaire, Generalized Anxiety Disorder scale (GAD-7), Patients Health Questionnaire depression scale (PHQ-9), Pregnancy Pressure Scale (stocktickerPPS), Perceived Social Support Scale (PSSS), General Self-Efficacy Scale (GSES), and Connor-Davidson Resilience scale (CD-RISC). We used the Amos2.03 system to build a structural equation model.

**Results:**

A total of 774 subjects had an average age of 30 years and an average gestational age of 23 weeks. Among the 774 respondents, 122 (15.8%) had moderate or above pregnancy stress (stocktickerPPS > 1), 376 (48.6%) had mild or above anxiety symptoms (GAD-7 ≥ 5), 456 (58.9%) had mild or above depression symptoms (PHQ-9 ≥ 5), 740 (95.6%) had moderate or above social support scores (PSSS ≥ 37), and 124 (16.0%) had good or above psychological resilience scores (CD-RISC ≥ 60). Notably, 372 (48.1%) people had a self-efficacy score above the overall average (GSES ≥ 2.6). Pregnancy stress had positive correlations with anxiety and depression (β = 0.57, 0.30, *P* < 0.01) and negative correlations with self-efficacy (β = −0.19, *P* < 0.01). Anxiety had positive correlations with depression (β = 0.54, *P* < 0.01) and negative correlations with social support (β = −0.45, *P* < 0.01). Social support had positive correlations with self-efficacy and resilience (β = 0.37, 0.47, *P* < 0.01). Resilience had negative correlations with anxiety (β = −0.09, *P* < 0.01), and self-efficacy had positive correlations with resilience (β = 0.41, *P* < 0.01).

**Conclusion:**

Identification of pregnancy stress should be emphasized in pregnant women with negative emotions. Efforts to strengthen the positive psychological qualities of pregnant women should focus on cultivating psychological resilience to reduce the occurrence of anxiety, and improving social support should be a priority because it can enhance psychological resilience and self-efficacy. We provide a reason to intervene in the negative emotions of pregnant women from the perspective of the positive psychology of pregnant women.

## Introduction

Women experience psychological and physical changes during pregnancy, and psychological problems occur frequently (Brunton et al., 2022). Hormone fluctuations in the body during pregnancy can lead to emotional instability, such as anxiety, depression, tension, and other negative emotions. In psychology, anxiety, tension, anger, depression, sadness, pain, and other emotions are collectively referred to as negative emotions. A growing number of studies have shown that the prevalence of prenatal anxiety in Chinese pregnant women is high (7.9–68.4%) (Hou et al., 2018; Tang et al., 2019). Furthermore, the prevalence of prenatal anxiety was reported to be 15.2–49.0% in several studies in Turkey, South Africa, Pakistan, Australia, Canada, the United Kingdom, and the United States (Howard et al., 2014; Waqas et al., 2015; Dennis et al., 2017). In recent years, because of the outbreak of COVID-19, many researchers have paid attention to the mental health of pregnant women, especially anxiety and depression (Ramiro-Cortijo et al., 2021). Numerous studies have focused on assessing anxiety, depression, and other psychological disorders, exploring their related risk factors and understanding pregnancy-related diseases and adverse pregnancy outcomes (García-Blanco et al., 2018).

Researchers have proposed various strategies to alleviate prenatal anxiety and depression in pregnant women. The positive psychology approach involves the treatment of mental illness while also striving to stimulate and cultivate positive psychological qualities (An and Zeng, 2020; Abbasi et al., 2022). Methods are needed to assess the psychological problems of pregnant women in a timely manner, identify the factors that influence negative emotions, and help patients develop positive psychological qualities and improve their mental health (Guriganati et al., 2022).

Positive psychological qualities include social support, self-efficacy, resilience, and mindfulness level. Many previous studies have shown that self-efficacy is the closest determinant of a critical structure and behavior. Self-efficacy affects the physical and psychological state of the mother, such as anxiety and depression (Cramp and Brawley, 2009; Sónia et al., 2018). Self-efficacy can also help individuals cope with stress rationally and positively (Anca et al., 2012). Resilience is another positive psychological resource for preventing mental disorders. It is a dynamic process that enables people at any stage of life to cope with adversity, bounce back after hardship, deal with unpleasant feelings, and adapt to changes (Gagnon and Stewart, 2014; Jin et al., 2021). Pregnant women’s depression is affected by many factors. Social support is one of the important factors and has attracted the attention of many scholars. Social support is an individual’s perception of external support, which is negatively correlated with depressive symptoms of pregnant women, that is, the higher the level of social support, the milder the depressive symptoms during pregnancy (Behmard et al., 2022).

As a new type of nursing intervention, positive psychological counseling mainly promotes the psychological balance of patients by enhancing the communication between nurses and patients so as to improve the effectiveness of treatment, thereby improving the pregnancy outcome and the quality of life of patients (Li et al., 2020). Positive psychological qualities such as resilience, self-efficacy, and social support can be cultivated to improve the negative emotions of pregnant women, which in turn can improve the individual’s physical and mental positive status (Wu et al., 2017; Rastad et al., 2021). In this study, we evaluated 774 pregnant women to study the correlations between positive psychological qualities and prenatal negative emotions and to explore the factors that influence these characteristics.

## Materials and methods

### Study participants

From August 2021 to April 2022, we used a simple sampling method to study pregnant women who were treated at the Obstetrics and Gynecology Department of a tertiary hospital in Urumqi, Xinjiang. Inclusion criteria were a clear diagnosis of pregnancy, informed consent, and willingness to cooperate with researchers to complete the questionnaires. Exclusion criteria were no informed consent; incomplete data collection; severe mental, cognitive, hearing, or language communication disorders; severe pregnancy complications (Such as gestational diabetes and hypertension); and high-risk pregnancies (previous habitual abortion, fetal malformation, pelvic abnormalities, etc.). A total of 800 questionnaires were distributed, and 774 valid questionnaires were returned. The completion rate was 96.7%.

## Measures

### Demographic questionnaire

The general demographic questionnaire included general demographic data such as maternal age, education level, monthly family income, place of residence, and occupation. Other factors included maternal family environment and living conditions, marital relations, living conditions, exercise, psychological preparation for pregnancy, and pregnancy knowledge.

### Generalized anxiety disorder-7 (GAD-7) scale

A generalized anxiety scale was developed by Spitzer et al. The GAD-7 consists of seven items, each of which is scored on a scale of 0-3, and the total score ranges from 0 to 21. An overall score of 0-4 indicates normal; 5-9 indicates mild anxiety; 10-14 indicates moderate anxiety, and 15-21 indicates severe anxiety. This study uses the Chinese version of this questionnaire, which has been verified by domestic research, indicating that the questionnaire has good reliability and validity in Chinese pregnant women (Jiang et al., 2021). In this study, Cronbach’s α for the scale was 0.885.

### Patient health questionnaire depression scale-9 item (PHQ-9)

The PHQ-9 is a simple and effective self-rating assessment based on nine symptoms of depressive disorder. It has good reliability and validity in the auxiliary diagnosis of depressive disorder and in evaluating the severity of emotional symptoms. The scale used is 0 (absolutely not) to 3 (almost daily), and the total score ranges from 0 to 27. A score of 0-4 indicates no depressive symptoms; 5-9 indicates mild depressive symptoms; 10-14 indicates moderate depressive symptoms; 15-19 indicates moderate to severe depressive symptoms; and 20-27 indicates severe depressive symptoms (Chen et al., 2020; Zhang and Huang, 2022). The research and systematic review of relevant scholars at home and abroad have also confirmed that the Chinese version of the scale is widely used for screening depression in hospitalized patients, people in primary communities, and specific populations (Hinz et al., 2016; Sun et al., 2017). In this study, Cronbach’s α for the scale was 0.852.

### Pregnancy pressure scale

The PPS contains three dimensions with a total of 30 entries. Dimension 1 (parent role) contains 15 items (items 0-15), dimension 2 (mother and child health and safety) contains 8 items (16-23), and dimension 3 (body shape and physical activity change) contains 4 entries (24-27); three other items were not included in the dimension as other factors, namely, the last three items of the scale (28-30). The Likert 4 grading method is used to score the inventory, and the scale score is the actual total score of the scale divided by the total items of the scale (Chen et al., 2021). A score of 0 indicates no pressure; 0.001-1 indicates mild pressure; 1.001-2 indicates moderate pressure; and 2.001-3 indicates severe pressure (Kim and Chung, 2018). In this study, Cronbach’s α for the scale was 0.953.

### Perceived social support scale

The PSSS translated by Jiang Qianjin into Chinese version has 12 items, consisting of three dimensions, namely, family support, friend support, and other support. Each item is scored from 1 to 7 points ranging from strongly agree to strongly disagree. A total score of 12–36 indicates low-level support; 37–60 indicates medium-level support; and 61–84 indicates high-level support. A higher score indicates a higher level of perceived social support. PSSS has good reliability and validity, and its internal consistency coefficient is 0.93. The internal consistency reliability of family support in the subscale is 0.83, and the internal consistency reliability of friend support and other support is 0.82 and 0.76, respectively, which has reached the standard of psychometrics (Luo et al., 2020; Qi et al., 2020). In this study, Cronbach’s α of the scale was 0.97.

### General self-efficacy scale

We used the Chinese version of the GSES translated by Wang et al. in 2000 (Zhang, 2020). The scale consists of 10 items related to the self-confidence of individuals when they encounter setbacks or difficulties. For each item, the participants answered “completely incorrect,” “somewhat correct,” “mostly correct,” or “completely correct” according to their actual situation, which corresponded to 1 point, 2 points, 3 points, and 4 points, respectively. The total score ranged from 10 to 40 points. A higher total score indicates stronger self-efficacy. This scale has good reliability and validity when employed in the Chinese population. In this study, Cronbach’s α of the scale was 0.952.

### Connor–Davidson resilience scale

The CD-RISC includes three dimensions of tenacity (13 items), strength (8 items), and optimism (4 items) (Kuiper et al., 2019), and 0-4 points are assigned to answers of “completely different,” “rarely,” “sometimes,” “often,” and “almost always.” The total score on the scale was 0-100 points. A score < 60 indicates poor resilience; 60-70 indicates middle-level resilience; 70–80 indicates good resilience; and = 80 indicates excellent resilience (Li, 2020; Li et al., 2020). In this study, Cronbach’s α of the scale was 0.972.

### Statistical methods

We analyzed the data using SPSS version 25.0 (IBM, Armonk, NY, USA), and the count data are expressed as the number of cases and percentages. The measurement data that obeyed the normal distribution were statistically described. The data conformed to normality as detected by the Shapiro–Wilk test (*P* > 0.05). Scores for the six scales were analyzed using Pearson’s correlation analysis. We used the IBM SPSS Amos23.0 modeling and analysis system to create the path analysis diagram of the relationship between the factors. The final path analysis model was obtained through continuous correction of the degree of model fitting. The criterion of structural equation model fit evaluation index is goodness of fit index (GFI) > 0.9, adjusted goodness of fit index (AGFI) > 0.9, PGFI > 0.5, IFI > 0.9, Tucker-Lewis coefficient (TLI) > 0.9, comparative fit index (CFI) > 0.9, CMIN/DF < 5, root mean square error of approximation (RMSEA) < 0.05 (Good fit), or RMSEA < 0.08 (reasonable adaptation). *P* < 0.05 indicated that differences were statistically significant.

## Results

### General demographic data

Among the 774 pregnant women included in this study, 686 (88.6%) were Han and 88 (11.4%) were ethnic minorities. Of the participants, 60 (7.8%) were younger than 25 years old, 638 (82.4%) were between 25 and 35 years old, and 76 (9.8%) were older than 35 years old. Sixty-eight women (8.8%) had an education level below high school, 216 (27.9%) women attended high school and college, 395 (51.0%) women had a bachelor’s degree, and 95 (12.3%) women had a master’s degree or above. Of the participants, 558 (72.1%) were employed and 216 (27.9%) were housewives. Notably, 45 (5.8%) women lived in rural areas and 729 (94.2%) lived in urban areas.

### Positive psychological scores

In this study, among the 774 respondents, 740 (95.6%) had moderate or above social support scores (PSSS ≥ 37), 124 (16.0%) had good or above psychological resilience scores (CD-RISC ≥ 60), and 372 (48.1%) had a self-efficacy score above the overall average (GSES ≥ 2.6). The average PSSS score was 63.29 ± 14.19 (in the middle of the range); the average GSES score was 26.18 ± 7.25 (in the middle of the range); and the CD-RISC score was 41.10 ± 22.79 (poor level of psychological resilience) (Table 1). Pregnant women of different ages and with different levels of education and family income differed significantly in their GSES and CD-RISC scores. The social support level of pregnant women with different education levels, family income levels, and occupations was statistically significant.

**TABLE 1:** Demographic characteristics (*N* = 774).

Variable	Number (n)	Percent (%)
**Nationality**		
Ethnic Han	686	88.6
Minority	88	11.4
Age		
≤25	60	7.8
26∼35	638	82.4
>35	76	9.8
**Education**		
Lower than high school	68	8.8
High school or technical secondary	216	27.9
Bachelor	395	51.0
Master	95	12.3
**Monthly family income**		
≤3,000	34	4.4
3,001∼5,000	185	23.9
5,001∼8,000	214	27.6
>8,000	341	44.1
**Occupation**		
Full-time job	558	72.1
Housewives	216	27.9
**Living site**		
Village	45	5.8
City	729	94.2

### Negative emotion scores

Among the 774 respondents, 122 (15.8%) had moderate or above pregnancy stress (PPS > 1), 376 (48.6%) had mild or above anxiety symptoms (GAD-7 ≥ 5), and 456 (58.9%) had mild or above depression symptoms (PHQ-9 ≥ 5). Table 2 shows that of the 774 pregnant women evaluated in this study, the GAD-7 score was 4.70 ± 3.54, and 376 (48.6%) participants had anxiety symptoms. The average PHQ-9 score was 6.11 ± 4.14, and 456 (58.9%) women had depressive symptoms. The mean PPS score was 0.56 ± 0.45, and 122 (15.8%) patients had pregnancy stress. The anxiety score differed significantly among pregnant women with different educational levels, as did the depression status of participants with different educational levels and occupations. Pregnancy pressure differed significantly between women with or without an occupation and between different living sites.

**TABLE 2:** The positive psychological level of the pregnant women assessed in this study.

Variable	GSES (Mean ± SD)	CD-RISC (Mean ± SD)	PSSS (Mean ± SD)
**Nationality**			
Ethnic Han	26.15 ± 7.28	41.22 ± 22.69	63.33 ± 14.22
Minority	26.44 ± 6.98	40.16 ± 23.64	62.93 ± 14.00
*t*	−0.359	0.412	0.252
*P*	0.639	0.332	0.396
**Age**			
≤25	22.42 ± 7.37	31.85 ± 20.79	59.18 ± 17.86
26∼35	26.37 ± 7.03	41.34 ± 22.42	63.52 ± 13.73
>35	27.57 ± 8.05	46.37 ± 25.41	64.58 ± 14.48
*F*	9.931	7.123	2.925
*P*	0.000	0.001	0.054
**Education**			
Lower than high school	21.63 ± 7.61	25.57 ± 19.82	50.93 ± 16.39
High school or technical secondary	25.89 ± 7.14	38.76 ± 21.91	62.06 ± 13.71
Bachelor	26.82 ± 7.13	44.06 ± 22.89	64.92 ± 13.44
Master	27.47 ± 6.49	45.23 ± 21.32	68.15 ± 11.36
*F*	11.519	15.363	25.387
*P*	0.000	0.000	0.000
**Monthly family income**			
≤3,000	23.82 ± 8.49	31.06 ± 25.87	53.74 ± 19.63
3,001∼5,000	25.33 ± 7.24	37.09 ± 21.80	61.15 ± 14.05
5,001∼8,000	25.21 ± 6.57	38.11 ± 19.70	62.29 ± 13.06
>8,000	27.49 ± 7.35	46.16 ± 23.77	66.04 ± 13.68
*F*	7.240	11.383	11.621
*P*	0.000	0.000	0.000
**Occupation**			
Full-time job	26.72 ± 7.18	42.89 ± 22.36	64.71 ± 13.38
Housewives	24.80 ± 7.24	36.49 ± 23.29	59.61 ± 15.54
*t*	3.332	3.532	4.538
*P*	0.878	0.310	0.000
**Living site**			
Village	24.22 ± 8.93	32.29 ± 24.30	55.91 ± 18.00
City	26.30 ± 7.12	41.65 ± 22.59	63.74 ± 13.81
*t*	−1.873	−2.684	−3.622
*P*	0.042	0.638	0.017

### Correlation between positive psychological qualities and negative emotions of pregnant women

We detected significant positive correlations in pairwise comparison between prenatal anxiety, depression, and pregnancy stress. For the positive psychological qualities, comparisons of self-efficacy, resilience, and social support revealed significant positive correlations. We found a significant negative correlation between negative emotions and positive psychological qualities of pregnant women (Table 3).

**TABLE 3:** Negative emotion score of pregnant women.

Variable	GAD-7 (Mean ± SD)	PHQ-9 (Mean ± SD)	PPS (Mean ± SD)
**Nationality**			
Ethnic Han	4.71 ± 3.51	6.13 ± 4.15	0.56 ± 0.45
Minority	4.59 ± 3.76	6.01 ± 4.07	0.59 ± 0.42
*t*	0.304	0.249	−0.576
*P*	0.530	0.993	0.715
**Age**			
≤25	5.73 ± 3.74	6.95 ± 4.87	0.68 ± 0.47
26∼35	4.60 ± 3.39	6.05 ± 3.92	0.56 ± 0.43
>35	4.74 ± 4.42	6.04 ± 5.18	0.50 ± 0.53
*t*	2.848	1.324	2.855
*P*	0.059	0.267	0.058
**Education**			
Lower than high school	6.16 ± 4.18	7.00 ± 5.14	0.65 ± 0.56
High school or technical secondary	4.93 ± 3.45	6.54 ± 4.21	0.55 ± 0.46
Bachelor	4.41 ± 3.39	5.79 ± 3.97	0.56 ± 0.43
Master	4.34 ± 3.58	5.86 ± 3.78	0.55 ± 0.38
*F*	5.478	2.718	0.942
*P*	0.001	0.044	0.420
**Monthly family income**			
≤ 3,000	5.41 ± 2.87	6.06 ± 3.97	0.55 ± 0.44
3,001∼5,000	4.98 ± 3.53	5.99 ± 4.15	0.52 ± 0.41
5,001∼8,000	4.81 ± 3.58	6.36 ± 3.93	0.62 ± 0.47
>8,000	4.41 ± 3.56	6.03 ± 4.29	0.55 ± 0.45
*F*	1.689	0.361	2.070
*P*	0.168	0.781	0.103
**Occupation**			
Full-time job	4.50 ± 3.41	5.83 ± 3.88	0.54 ± 0.42
Housewives	5.21 ± 3.80	6.84 ± 4.68	0.62 ± 0.50
*t*	−2.547	−3.058	−2.166
*P*	0.179	0.005	0.009
**Living site**			
Village	6.18 ± 3.77	6.71 ± 4.89	0.54 ± 0.44
City	4.61 ± 3.50	6.08 ± 4.09	0.56 ± 0.45
*t*	2.904	0.995	−3.159
*P*	0.453	0.183	0.002

### Correlation path analysis of positive psychology and negative emotion of pregnant women

**FIGURE 1:**
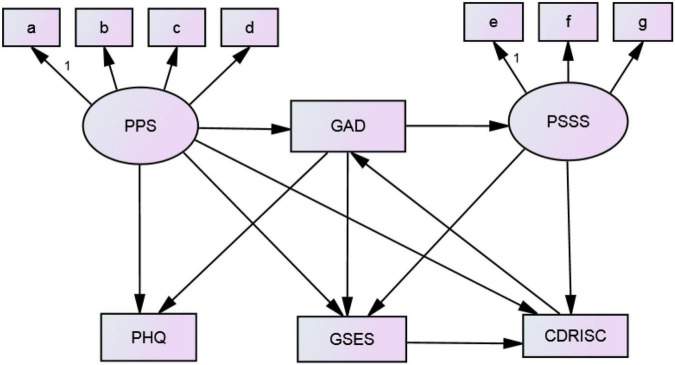
Theoretical model of path analysis of positive psychology and negative emotion of pregnant women.

According to the previous research results of correlation analysis, path analysis relationship is found between positive psychology and anxiety, depression, and other factors in pregnant women. Combining relevant literature, expertise, and results of single factor analysis and correlation analysis builds the initial theoretical model (refer to Figure 1).

**FIGURE 2:**
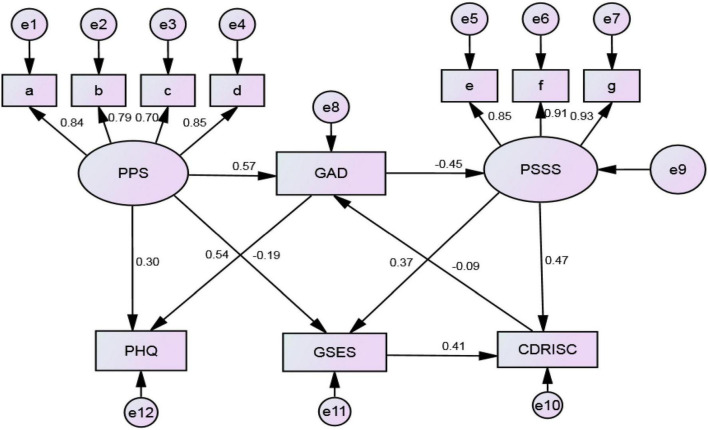
Analysis of positive and negative emotional paths of the 774 pregnant women evaluated in this study. PPS, pregnancy pressure scale; GAD, generalized anxiety disorder scale; PSSS, perceived social support scale; PHQ, patient health questionnaire depression scale; GSES, general self-efficacy scale; CD-RISC, Connor–Davidson resilience scale; a, parent role; b, mother and child health; c, body shape; d, others; e, family support; f, friend support; g, others support.

The path analysis results (Table 4) showed the path relationships among anxiety, depression, pregnancy stress, social support, self-efficacy, and psychological resilience in the 774 participants in this study. After using Amos23.0 to construct the initial theoretical model, we obtained the structural equation model by continuously adjusting and revising the model. The model had an acceptable degree of fit and was statistically significant (*P* < 0.001). The fit indices are as follows: CMIN/DF = 4.803, RMSEA = 0.070, GFI = 0.959, AGFI = 0.930, CFI = 0.959, and TLI = 0.960. Figure 2 shows the path analysis diagram.

**TABLE 4:** Correlation analysis of positive psychology and negative emotion of pregnant women.

Variable	Prenatal anxiety	Depression	Pregnancy stress	Self-efficacy	Resilience	Social support
Prenatal anxiety	1	−	−	−	−	−
Depression	0.698**	1	−	−	−	−
Pregnancy stress	0.509**	0.556**	1	−	−	−
Self-efficacy	-0.236**	-0.256**	-0.312**	1	−	−
Resilience	-0.347**	-0.367**	-0.406**	0.606**	1	−
Social support	-0.384**	-0.387**	-0.371**	0.416**	0.605**	1

**P < 0.01 (double tails), the correlation was significant.

The results of the path analysis (Table 5) showed that pregnancy pressure has a positive influence on anxiety and depression (β = 0.57, 0.30) and a negative influence on self-efficacy (β = −0.19). Anxiety has a positive influence on depression (β = 0.54) and a negative influence on social support (β = −0.45). Meanwhile, social support has a positive influence on self-efficacy and resilience (β = 0.37, 0.47). Resilience has a negative influence on anxiety (β = −0.09), and self-efficacy has a positive influence on resilience (β = 0.41).

**TABLE 5:** Path analysis results of positive psychology and negative emotion of pregnant women.

Path	β	S.E.	C.R.	*P*
Anxiety ← Pregnancy stress	0.569	0.028	15.207	<0.001
Self-efficacy ← Pregnancy stress	-0.194	0.054	-5.437	<0.001
Depression ← Pregnancy stress	0.299	0.028	9.226	<0.001
Depression ← Anxiety	0.538	0.035	17.999	<0.001
Social support ← Anxiety	-0.448	0.052	-10.646	<0.001
Self-efficacy ← Social support	0.374	0.060	10.334	<0.001
Resilience ← Social support	0.474	0.167	14.758	<0.001
Anxiety ← Resilience	-0.085	0.07	1.998	0.046
Resilience ← Self-efficacy	0.407	0.088	14.651	<0.001

## Discussion

In this study, we investigated the mental health of pregnant women from the perspective of positive psychological qualities and negative emotions. We found that the social support and self-efficacy of pregnant women were at a medium level, but their psychological resilience was poor. The mental resilience value of pregnant women measured in our study is lower than that reported by Shang et al. (2019). We found that psychological resilience is related to the age, education level, and family income of pregnant women. As 36.8% of the pregnant women evaluated in this study had an education level lower than junior college, this factor may affect the overall level of psychological resilience. Other studies showed that resilience as a protective factor can have positive effects on individual physical and mental health (Wu et al., 2017; Luo et al., 2022). In contrast, poor psychological resilience can increase the risk of anxiety and depression in pregnant women. In this study, the incidence of anxiety and depression was 48.6 and 58.9%, respectively. Meanwhile, in previous studies in Turkey, South Africa, and Pakistan, the prevalence of prenatal anxiety was 15.9, 23, and 49% (Van Heyningen et al., 2017; Kirupamani et al., 2019). Cultural clashes may exacerbate prenatal diseases such as stress and anxiety. This may explain the high rate of prenatal anxiety and depression among Chinese women.

In recent years, research on the mental health of pregnant women has mostly consisted of in-depth studies from the perspective of anxiety and depression, with less research from the perspective of positive psychological qualities (Corno et al., 2018). Therefore, we investigated both the negative emotions of pregnant women and their positive psychological qualities to understand the relationship between them. We found that positive psychological qualities were negatively correlated with the negative emotions of pregnant women. This result is consistent with previous research results (Zhao et al., 2019; Shaghaghi et al., 2020), which showed that positive psychological counseling can reduce the increase of negative psychological emotions of pregnant women, increase the cooperation of pregnant women with medical staff, and improve pregnancy outcomes (MacGinty et al., 2020). When women face the stressful event of pregnancy, they inevitably have concerns about themselves and the fetus, and these concerns are the direct influencing factors of pregnancy stress (Nakamura et al., 2018). Our results indicated that pregnancy stress had a direct influence on anxiety and depression, which is consistent with previous research results (Zhao et al., 2020). Therefore, reducing the psychological stress of women during pregnancy was an important factor in preventing prenatal depression.

Path analysis showed that two positive psychological qualities (social support and self-efficacy) had an indirect influence on anxiety and depression of pregnant women; they both affected depression by first regulating anxiety. This result reveals that anxiety is an important influencing factor for depression. Previous studies have reported that anxiety had the highest direct influence among the influencing factors of depression in pregnant women (Yang et al., 2020; Ma et al., 2021). Pregnancy stress, family income, psychological preparation for pregnancy, and social support of pregnant women all have an influence on depression through anxiety. However, psychological resilience has a direct influence on anxiety. Zou et al. (2022) showed that pregnant women with poor psychological resilience were more likely to have anxiety. Social support has a direct positive influence on self-efficacy and resilience, thus improving the social support of pregnant women will enhance these qualities. This study explores the relationship between the positive psychological level and the negative emotions of pregnant women and finds that paying attention to the negative emotions of pregnant women should also focus on the positive psychology of pregnant women. Cultivating the positive psychological level of pregnant women can buffer the occurrence of negative emotions of pregnant women.

Our study had certain limitations. First, we used a cross-sectional survey approach, and interventions need to be tested in future experiments and follow-up studies need to be conducted. Second, the sample size of this study was not large, so further studies with more participants from more hospitals are needed to provide data that are representative of the population of pregnant women in China. Nevertheless, our results provided a better understanding of the relationship between positive psychological qualities and negative emotions of pregnant women. We found that pregnancy stress had a direct influence on anxiety and depression and an indirect influence on social support and resilience; resilience had a direct influence on anxiety and an indirect effect on other negative emotions; and social support could directly influence self-efficacy and resilience. Therefore, clinicians should focus on pregnancy stress to alleviate the anxiety and depression of pregnant women. Cultivating resilience in pregnant women could greatly reduce the occurrence of anxiety, and improving social support could help cultivate psychological resilience and self-efficacy.

## Data availability statement

The raw data supporting the conclusions of this article will be made available by the authors, without undue reservation.

## Ethics statement

The studies involving human participants were reviewed and approved by Ethics Committee of Xinjiang Medical University (approval no.: XJYKDXR20220302029). The patients/participants provided their written informed consent to participate in this study.

## Author contributions

XT and GW contributed to the study’s conception and design. WZ contributed to the development of the material and data collection. XT wrote the manuscript. BS and XW assisted in the technical design of the survey. TJ helped with the data analysis and its processing in SPSS. All authors contributed to the article and approved the submitted version.
